# Quadruple Quorum-Sensing Inputs Control *Vibrio cholerae* Virulence and Maintain System Robustness

**DOI:** 10.1371/journal.ppat.1004837

**Published:** 2015-04-15

**Authors:** Sarah A. Jung, Christine A. Chapman, Wai-Leung Ng

**Affiliations:** 1 Department of Molecular Biology and Microbiology, Tufts University School of Medicine, Boston, Massachusetts, United States of America; 2 Program in Molecular Microbiology, Sackler School of Graduate Biomedical Sciences, Tufts University, Boston, Massachusetts, United States of America; Emory University School of Medicine, UNITED STATES

## Abstract

Bacteria use quorum sensing (QS) for cell-cell communication to carry out group behaviors. This intercellular signaling process relies on cell density-dependent production and detection of chemical signals called autoinducers (AIs). *Vibrio cholerae*, the causative agent of cholera, detects two AIs, CAI-1 and AI-2, with two histidine kinases, CqsS and LuxQ, respectively, to control biofilm formation and virulence factor production. At low cell density, these two signal receptors function in parallel to activate the key regulator LuxO, which is essential for virulence of this pathogen. At high cell density, binding of AIs to their respective receptors leads to deactivation of LuxO and repression of virulence factor production. However, mutants lacking CqsS and LuxQ maintain a normal LuxO activation level and remain virulent, suggesting that LuxO is activated by additional, unidentified signaling pathways. Here we show that two other histidine kinases, CqsR (formerly known as VC1831) and VpsS, act upstream in the central QS circuit of *V*. *cholerae* to activate LuxO. *V*. *cholerae* strains expressing any one of these four receptors are QS proficient and capable of colonizing animal hosts. In contrast, mutants lacking all four receptors are phenotypically identical to LuxO-defective mutants. Importantly, these four functionally redundant receptors act together to prevent premature induction of a QS response caused by signal perturbations. We suggest that the *V*. *cholerae* QS circuit is composed of quadruple sensory inputs and has evolved to be refractory to sporadic AI level perturbations.

## Author Summary

Quorum-sensing (QS) is a microbial cell-cell communication process that allows bacteria to function as a collective group. Many pathogens, including *Vibrio cholerae*, the causative agent of cholera, depend on QS to regulate important cellular processes that are essential for survival and adaptation inside and outside of their hosts. Since its discovery, the *V*. *cholerae* QS system has served as a model to understand how bacterial pathogens employ QS for temporal control of virulence factor production. Yet, after a decade of research, our understanding of the *V*. *cholerae* QS system is still incomplete. Here we re-define the QS network architecture of this important pathogen. We show that two novel sensory inputs function in parallel with the two canonical QS pathways to regulate *V*. *cholerae* virulence gene expression. Moreover, our study illustrates a strategy that bacteria employ to maintain QS system robustness. By perceiving multiple parallel sensory inputs, the *V*. *cholerae* QS network is structured to be highly resistant to signal perturbations, therefore preventing premature commitment to QS. Our study provides new insights into how bacterial pathogens integrate multiple sensory signals to elicit robust and coordinated QS responses.

## Introduction

Bacteria produce and detect multiple classes of chemical signals called autoinducers to monitor local population density and species complexity. This cell-to-cell communication process, called Quorum Sensing (QS), allows groups of bacteria to synchronize population-wide gene expression and effectively carry out collective behaviors that are presumably ineffective if performed by a single bacterial cell acting alone. Disruption of the QS signal transduction cascade leads to uncoordinated gene expression and renders many pathogenic bacteria avirulent [1–5].

**Fig 1:**
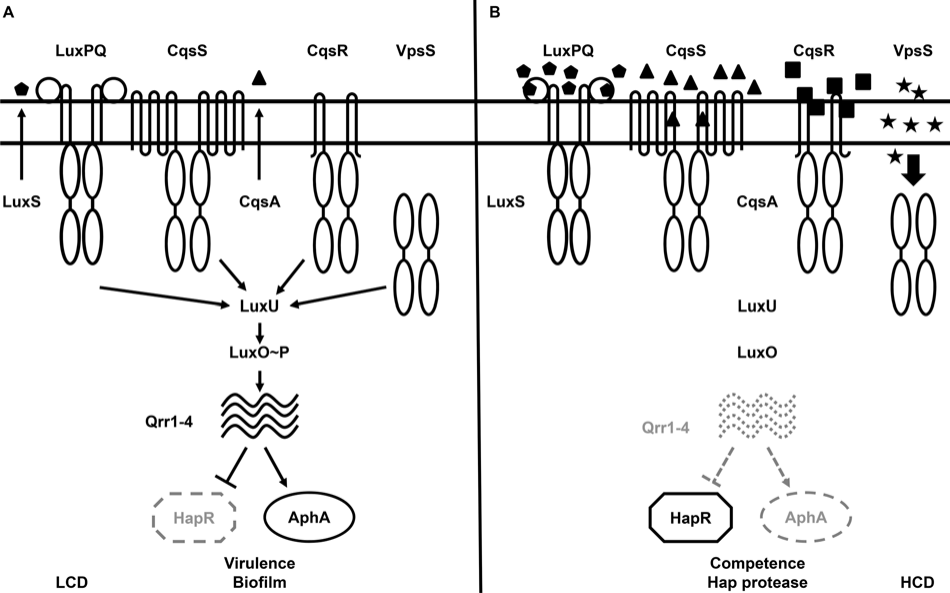
The proposed *Vibrio cholerae* quorum-sensing (QS) signal transduction system. (A) QS signal transduction at low cell density (LCD). Autoinducer levels are low and kinase activities of CqsS, LuxPQ, VpsS, and CqsR (VC1831) predominate. Through LuxU, these four histidine kinase receptors activate LuxO via phosphorylation. Activated LuxO promotes transcription of the Qrr1-4 small RNAs (sRNAs), which in turn activate AphA expression and repress HapR production. The LCD QS regulon includes genes required for virulence factor production and biofilm formation. (B) QS signal transduction at high cell density (HCD). Autoinducer levels are high and dephosphorylation activities of the four receptors predominate. At this condition, LuxO is dephosphorylated, Qrr1-4 sRNAs are not transcribed, and AphA expression is repressed while HapR protein is produced. Solid and dotted lines denote regulatory factors that are produced and not produced, respectively. Mutants lacking the four receptors altogether phenotypically mimic the HCD QS state.

*Vibrio cholerae*, the etiological agent of the diarrheal disease cholera, uses QS to regulate virulence factor production, biofilm formation, Type VI secretion, and competence development, all of which are important for survival and adaptation inside and outside of its human hosts [6–17]. Two parallel QS signaling systems that function via phosphorelay-type regulatory pathways have been identified in *V*. *cholerae* [6]. The CqsA/CqsS system, which produces and detects CAI-1 (*S*-3-hydroxytridecan-4-one) as a QS signal, is present in many *Vibrio* species and is believed to be used for intra-genus communication [18–23]. The LuxS/LuxPQ system, which produces and detects AI-2 (*S*-TMHF-borate) as a QS signal, is present in many bacterial species and is believed to be used for inter-species signaling [6, 24–27]. In environments where the concentrations of these two autoinducers are below their detection threshold, such as at low cell density (LCD), CqsS and LuxQ function as kinases. They hydrolyze ATP and shuttle the phosphoryl group, via a histidine-phosphotransfer protein LuxU, to the key response regulator, LuxO. Phosphorylated LuxO (LuxO~P) activates transcription of the genes encoding four regulatory sRNAs, called Qrr1-4 [28]. Aided by the RNA chaperone Hfq, Qrr1-4 activate the translation of the AphA regulator and inhibit the translation of the HapR regulator (Fig 1A) [6, 28–30].

At high cell density (HCD), when autoinducers accumulate to high levels, the kinase activities of CqsS and LuxQ are inhibited by binding of their cognate signals. As a consequence, phosphate flow is reversed, leading to dephosphorylation and deactivation of LuxO. Transcription of *qrr*1-4 terminates and, hence, HapR, but not AphA, is produced (Fig 1B) [6, 28–30]. Reciprocal production of AphA and HapR at LCD and HCD is central to the switch from individual to group behaviors in *Vibrio* species [29]. Together these two transcriptional regulators regulate the expression levels of over 100 genes [7, 29].

Although CqsS and LuxQ both contribute to LuxO activation (Fig 1), strikingly, mutants missing both receptors are phenotypically identical to the wild-type and remain virulent [6]. Thus, additional unknown signaling pathways are predicted to activate LuxO [6, 31]. A clue to the identity of a potential LuxO-activation pathway came from a study in which overexpression of a hybrid histidine kinase VpsS leads to a LuxO-dependent up-regulation of the biofilm biosynthetic gene *vpsL* in *V*. *cholerae*, suggesting that VpsS could regulate LuxO activity to control biofilm formation [32]. Moreover, the purified receiver domains of VpsS and another hybrid histidine kinase VC1831 are capable of effectively accepting the phosphoryl group from phosphorylated LuxU *in vitro* [32]. However, it is unclear if VpsS and VC1831, together with CqsS and LuxQ, function as phosphoryl group donors to activate LuxO and control QS inside *V*. *cholerae* cells. Furthermore, it is also unknown if, and to what extent, each of these four histidine kinase receptors is individually contributing to the global control of the QS response in *V*. *cholerae*. The role of VpsS and VC1831 in *V*. *cholerae* pathogenesis also has not been investigated.

Here we report the connections between VpsS and CqsR and QS in *V*. *cholerae* (VC1831 is renamed as CqsR hereafter based on its role as Cholera Quorum Sensing Receptor). VpsS and CqsR function in parallel with CqsS and LuxQ and act upstream of LuxO in the *V*. *cholerae* QS signal transduction pathway. Indeed *V*. *cholerae* is capable of QS when any single one of these four receptors is present. Furthermore, in addition to CAI-1 and AI-2, additional stimuli whose levels presumably vary depending on cell density, are perceived by VpsS and CqsR to modulate QS. Finally, multiple functionally redundant receptors that control a signal regulator (i.e., LuxO) enable a QS response that is insensitive to perturbations in the cognate sensory cues.

## Results

### *V*. *cholerae* host colonization requires LuxO activation and Qrr sRNAs

**Fig 2:**
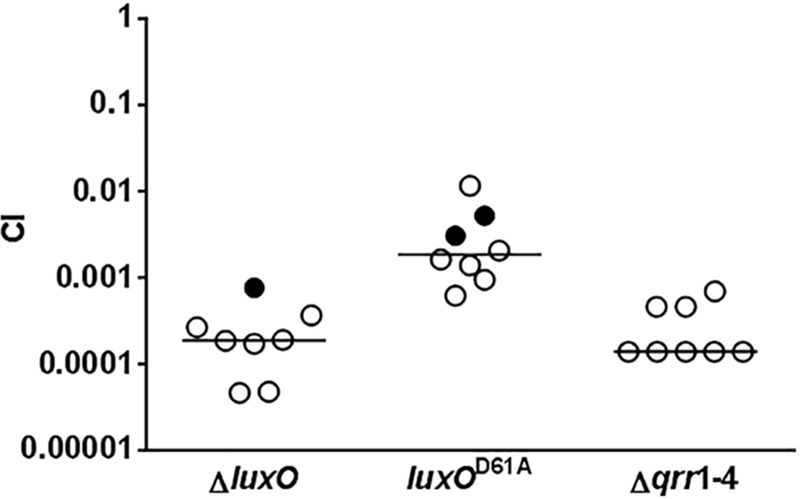
Activated LuxO and Qrr1-4 sRNAs are required for *V*. *cholerae* host colonization. Competitive indices (CI) were determined between wild-type Δ*lacZ* and the indicated *V*. *cholerae* mutants in infant mice 24 hr post-infection. Each symbol represents the CI in an individual mouse and horizontal lines indicate the median for each competition. Open symbols represent data below the limit of detection for the mutant strain. In that case, it was assumed that there was 1 mutant CFU present at the next lowest dilution of the wild-type sample to calculate the CIs.

Previous studies established that LuxO, the key response regulator in the *V*. *cholerae* QS system, is activated by phosphorylation at the conserved Asp61 by CqsS and LuxQ at LCD (Fig 1) [33]. *V*. *cholerae* mutants lacking LuxO are unable to express Qrr1-4 sRNAs; as a result, they fail to express AphA and instead produce HapR at all population densities [6, 28]. Therefore, Δ*luxO* mutants are highly attenuated in colonization of animal hosts [6, 7]. Surprisingly, *V*. *cholerae* mutants lacking both CqsS and LuxQ are phenotypically identical to the wild-type and colonize animal hosts effectively. Thus, LuxO appears to be activated by additional mechanisms [6]. Alternatively, these results could be interpreted to mean that unphosphorylated LuxO, but not phosphorylated LuxO, is required for host colonization and there is no additional source of activation. If the former model is correct, a *V*. *cholerae luxO* ^D61A^ mutant, expressing a form of LuxO that is incapable of being phosphorylated, should be defective in colonizing animal hosts. Indeed, both Δ*luxO* and *luxO* ^D61A^ mutant cells were out-competed by the wild-type in an infant mouse colonization model, however, there was a 10-fold difference in the CIs observed for these two mutants. In many cases, we did not detect any Δ*luxO* and *luxO* ^D61A^ mutants inside the animal hosts (Fig 2). Furthermore, *V*. *cholerae* cells lacking all 4 Qrr sRNAs, the only known targets of activated LuxO, were defective in host colonization (Fig 2). Together, these *in vivo* data indicate that phosphorylated LuxO and the downstream Qrr sRNAs are required for *V*. *cholerae* host colonization, and further suggest that phosphorylation by CqsS and LuxQ are not the only sources of LuxO activation.

### LuxO activation depends on LuxU only

**Fig 3:**
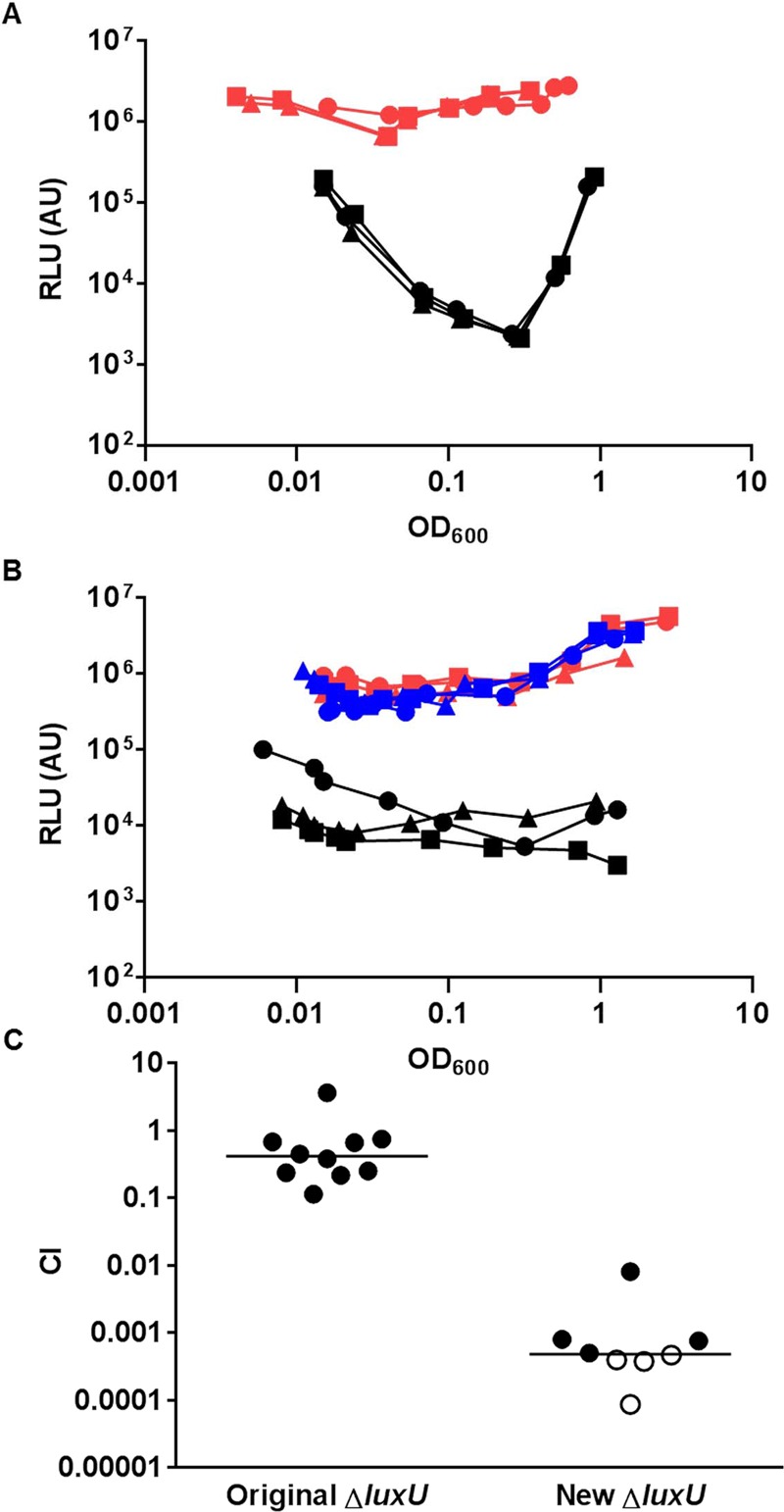
LuxU is the key HPT protein in the *V*. *cholerae* QS signal transduction system. (A-B) The QS response in different *V*. *cholerae* mutants missing LuxU was measured with a HapR-dependent bioluminescence operon. Normalized light production was measured in different Δ*luxU* mutants in triplicates. RLU denotes relative light units. (A) Black lines and symbols represent the original Δ*luxU* mutant (WN3045). Red lines and symbols represent the new Δ*luxU* mutant (WN3557). (B) Blue lines and symbols represent the new Δ*luxU* strain with an empty vector. Red lines and symbols represent the new Δ*luxU* strain expressing WT *luxO*. Black lines and symbols represent the new Δ*luxU* strain expressing *luxO* ^G333S^. (C) Effect of different Δ*luxU* mutations in *V*. *cholerae* infections. Competitive indices (CI) were determined between WT Δ*lacZ* and the indicated *V*. *cholerae* mutants in infant mice 24 hr post-infection. Each symbol represents the CI in an individual mouse and horizontal lines indicate the median for each competition. Open symbols represent data below the limit of detection for the mutant strain.

To further explore the pathway for LuxO activation, we focused on the protein that interacts with LuxO in the *V*. *cholerae* QS circuit. Only a single histidine phosphotransfer (HPT) protein, LuxU, is known to interact with LuxO and link to LuxO activation ([6], Fig 1). Unexpectedly, *V*. *cholerae* mutants lacking LuxU were shown to be active in QS gene regulation and colonize animal hosts effectively [6]. These findings seem to contradict the apparent role of active LuxO in *V*. *cholerae* pathogenicity regulation (Fig 2). Alternatively, LuxO could be activated by interacting with other HPT proteins. However, LuxU was found to be required for *V*. *cholerae* virulence in two independent genome-scale transposon mutant analyses [34, 35]. To resolve these conflicting results, we revisited the role of LuxU in *V*. *cholerae* QS control and pathogenicity regulation. We constructed a new Δ*luxU* mutant (WN3557) and compared its QS response to that of the Δ*luxU* mutant (WN3045) previously reported [6]. We used the heterologous *Vibrio harveyi luxCDABE* luciferase operon to measure QS-dependent gene regulation, because expression of this operon is activated by HapR, whose level is inversely proportional to the amount of activated LuxO inside the cell ([6], Fig 1). Therefore, if LuxU is the major HPT protein that is essential for LuxO activation, mutants lacking LuxU would express a high level of luciferase and be bright. We found that bioluminescence production (shown as specific light production versus cell density) was very different between these two Δ*luxU* strains. The newly-constructed Δ*luxU* mutant cells were constitutively bright, indicating that LuxO is inactive and that HapR is constantly produced at all population densities (Fig 3A). In contrast, the original Δ*luxU* mutant cells were 10- to 100-fold darker, depending on the cell density at which they were sampled, indicating that less HapR is produced in the original Δ*luxU* mutant (Fig 3A). To understand these differences, we sequenced the *luxOU* locus of these Δ*luxU* strains. Using published *V*. *cholerae* genome sequences as a reference, we identified a missense mutation in *luxO* of the original Δ*luxU* strain, resulting in a change from glycine to serine at amino acid residue 333 of LuxO. In contrast, no mutation was identified in the *luxOU* locus of the new Δ*luxU* strain. To explain the difference in phenotype between the two strains, we hypothesized that LuxO^G333S^ mimics the active form of LuxO such that it does not require LuxU for activation. To test this hypothesis, plasmids expressing *luxO* ^*+*^ or *luxO* ^G333S^ were introduced into the new Δ*luxU* strain, and HapR-dependent bioluminescence from the resulting strains was measured. We found that extra copies of *luxO* ^*+*^ did not alter the specific bioluminescence production of the new Δ*luxU* mutant and the strain remained constitutively bright, similar to the empty plasmid control (Fig 3B). However, when *luxO* ^G333S^ was expressed in the new Δ*luxU* mutant, the resulting strain was >50-fold darker than the other two strains (Fig 3B). These results indicate that the *luxO* ^G333S^ mutation is dominant to the *luxO* ^*+*^ allele and is epistatic to the Δ*luxU* mutation. Although the activation mechanism is unclear, we suggest that LuxO^G333S^ mimics an active form of LuxO, bypassing the requirement of LuxU in QS signal transduction in the original Δ*luxU* strain. Consistent with the idea that LuxU is the key HPT protein in QS control, the new Δ*luxU* mutant cells were out-competed by the wild-type in the infant mouse colonization model, while the original Δ*luxU* cells were not (Fig 3C).

### VpsS and CqsR histidine kinases contribute to LuxO activation

**Fig 4:**
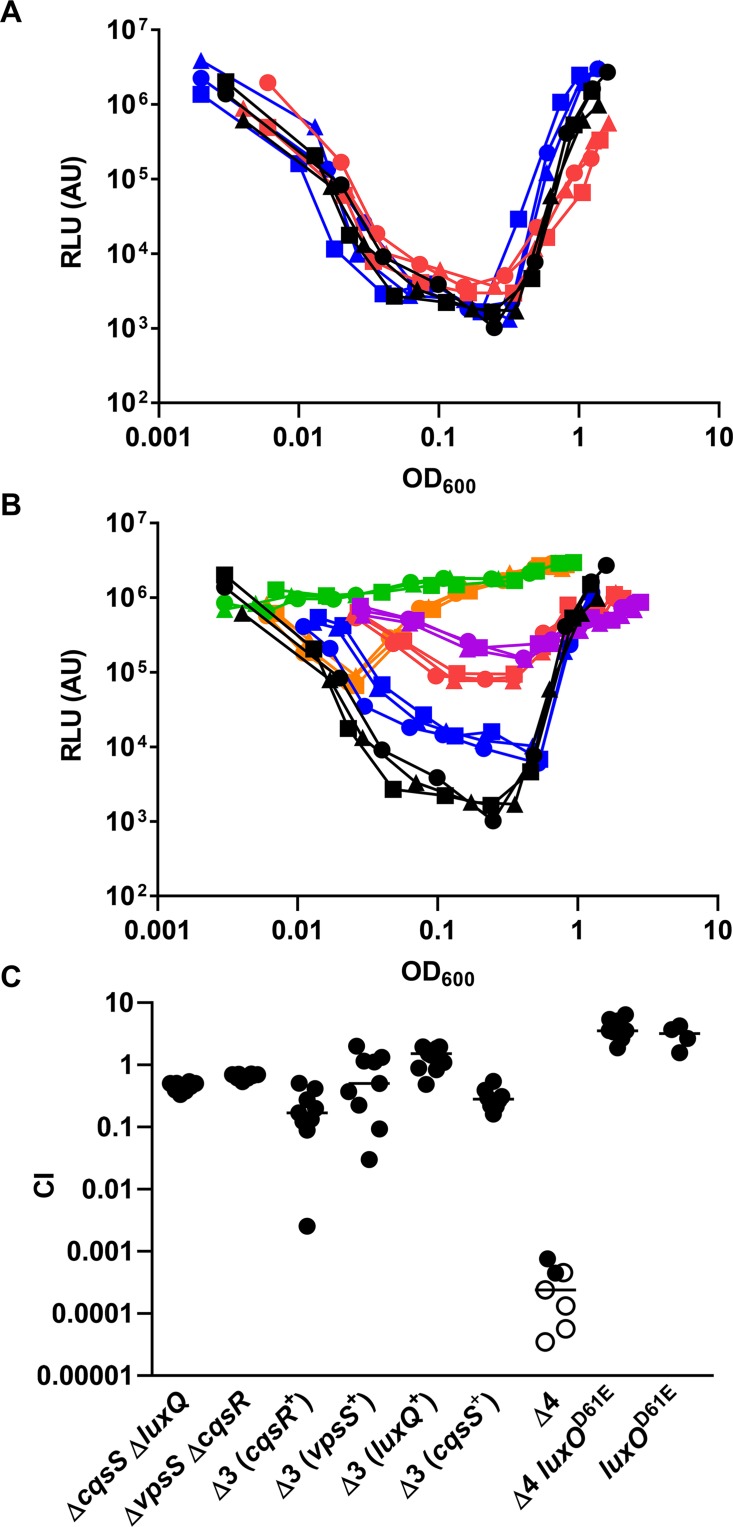
VpsS and CqsR contribute to the QS response in *V*. *cholerae*. (A-B) The QS response in different *V*. *cholerae* mutants missing multiple QS receptors was measured with a HapR-dependent bioluminescence operon. Normalized light production was measured in different receptor mutants in triplicates. RLU denotes relative light units. (A) Black lines and symbols represent the wild-type. Red lines and symbols represent the Δ*cqsS* Δ*luxQ* strain. Blue lines and symbols represent the Δ*vpsS* Δ*cqsR* strain. (B) Black lines and symbols represent the wild-type. Purple lines and symbols represent the Δ*cqsS* Δ*luxQ* Δ*vpsS* strain (CqsR only). Red lines and symbols represent the Δ*cqsS* Δ*luxQ* Δ*cqsR* strain (VpsS only). Orange lines and symbols represent the Δ*luxQ* Δ*vpsS* Δ*cqsR* strain (CqsS only). Blue lines and symbols represent the Δ*cqsS* Δ*vpsS* Δ*cqsR* strain (LuxQ only). Green lines and symbols represent the strain lacking all four receptors (Δ*cqsS* Δ*luxQ* Δ*vpsS* Δ*cqsR*). (C) Effect of receptor mutations in *V*. *cholerae* infections. Competitive indices (CI) were determined between wild-type Δ*lacZ* and the indicated *V*. *cholerae* mutants in infant mice 24 hr post-infection. Each symbol represents the CI in an individual mouse and the horizontal lines indicate the median for each competition. The open symbols represent data below the limit of detection for the mutant strain. Δ3 represents triple receptor mutants with the remaining receptor shown in parentheses. Δ4 represents a *V*. *cholerae* mutant missing all four receptors.

After confirming the importance of LuxU in LuxO activation, we hypothesized that, similar to CqsS and LuxQ, histidine kinases that employ LuxU as an intermediate phosphorelay partner are able to activate LuxO. The isolated receiver domains of two hybrid histidine kinases, VpsS and CqsR (VC1831), are capable of interacting with and effectively removing the phosphoryl group from phosphorylated LuxU *in vitro* [32]. Thus, we reasoned that full length VpsS and CqsR, when active, could phosphorylate LuxO via LuxU and contribute to its activation in *V*. *cholerae* cells. To explore these ideas, we measured HapR-dependent bioluminescence in mutants missing one or more of these histidine kinases. As previously shown [6], wild-type *V*. *cholerae* cells produce a U-shaped bioluminescence profile, representing the change in LuxO activity and HapR levels at different cell-densities (Fig 4A). At HCD (OD_600_ >1), *V*. *cholerae* produced a high level of HapR-dependent bioluminescence, indicating that LuxO activity is low in this condition (Fig 4A). When these HCD cells were diluted in fresh medium, specific luciferase activity was high initially since the enzyme had not been turned over from the overnight culture. However, when these diluted cells started to grow, HapR-dependent bioluminescence decreased due to activation of LuxO and repression of HapR production. Light production reached a minimum at OD_600_ ~0.5. Afterwards, HapR-dependent bioluminescence increased and reached a maximum at OD_600_ >1 (Fig 4A). Using the same assay, we found that cells missing both CqsS and LuxQ displayed a HapR-dependent bioluminescence profile indistinguishable from that of the wild-type, indicating that LuxO activation is still controlled by a cell density-dependent mechanism in the absence of these two known QS receptors (Fig 4A). Likewise, *V*. *cholerae* cells missing both VpsS and CqsR also displayed a HapR-dependent bioluminescence profile similar to that of the wild-type and the Δ*cqsS* Δ*luxQ* double QS receptor mutant (Fig 4A).

We constructed four different triple receptor mutants expressing only one of the four possible QS receptors and found that their HapR-dependent bioluminescence profiles were different from each other and from the wild-type (Fig 4B). Although each triple receptor mutant still displayed a U-shaped HapR-dependent bioluminescence profile, switching from low to high light production from LCD to HCD, these mutants all produced more light than the wild-type at LCD (Fig 4B). Mutant cells with only LuxQ showed the largest difference (~100-fold) in relative light production between LCD and HCD, while mutant cells with only CqsR showed the smallest difference (~10-fold). The temporal dynamics of the response in each triple receptor mutant were also distinct. Mutant cells expressing only CqsS switched from low to high light production at the lowest cell density (OD_600_ ~0.05), while the other three mutant strains switched at around the same cell density (OD_600_ ~0.5) (Fig 4B). Thus, our results indicate that CqsS, LuxQ, VpsS, and CqsR can each independently activate LuxO, but the influence of each receptor on the overall QS response is not identical.

We then measured the HapR-dependent bioluminescence in a Δ*luxQ* Δ*cqsS* Δ*vpsS* Δ*cqsR* quadruple receptor mutant and found that the profile was identical to the new Δ*luxU* mutant (Figs 3A and 4B), indicating that very little, if any, active LuxO is present. To determine if VpsS and CqsR both act upstream to activate LuxO, we introduced the *luxO* ^D61E^ mutation, which renders LuxO constitutively active by mimicking the phosphorylated form of the protein [8, 36], into the quadruple receptor mutant. We predicted that the *luxO* ^D61E^ allele would override the effect of the loss of all four histidine kinases. Indeed, we found that the Δ*luxQ* Δ*cqsS* Δ*vpsS* Δ*cqsR luxO* ^D61E^ strain was constitutively dark, similar to the *luxO* ^D61E^ mutant (S1 Fig). Likewise, LuxO activity could be partially restored when *cqsS*, *luxQ*, *vpsS*, or *cqsR* was individually overexpressed episomally in the quadruple receptor mutant (S2 Fig). Additionally, we used a *qrr*4-*lux* transcriptional fusion to study the contribution of CqsS, LuxQ, VpsS, and CqsR and found that each receptor alone was sufficient to support Qrr4 expression to different degrees at LCD (S3 Fig), while the quadruple receptor, Δ*luxO*, and new Δ*luxU* mutants all expressed very little, if any, Qrr4. As expected, the *luxO* ^D61E^ mutation restored Qrr4 expression in the quadruple receptor mutant (S3 Fig).

Previously, CsrA was proposed to enhance LuxO~P activity [31]. VpsS and CqsR could exert their regulatory effects on LuxO by modulating the activity of CsrA. However, a *csrA*::Tn5 insertion mutation that had been identified before was not sufficient to abolish Qrr4 production in the Δ*cqsS* Δ*luxQ* mutant (S4 Fig), arguing against the possibility that VpsS and CqsR signal through CsrA.

### A single QS receptor is sufficient to activate LuxO for host colonization

The above studies show that CqsS, LuxQ, VpsS, and CqsR each independently contributes to part of the QS response in *V*. *cholerae* growing under laboratory conditions. To determine the minimal requirement of LuxO activation through these histidine kinases that is sufficient for *V*. *cholerae* infection of animal hosts, we tested double, triple, and quadruple receptor mutants using an infant mouse colonization model. We found that the two double receptor mutants (Δ*luxQ* Δ*cqsS* and Δ*vpsS* Δ*cqsR*) and the four triple receptor mutants all colonized the small intestine effectively (Fig 4C). In contrast, the quadruple receptor mutant was highly defective in animal colonization (Fig 4C). While a slight advantage in host colonization (~2 fold) was observed for the *luxO* ^D61E^ mutants in the wild-type genetic background, the *luxO* ^D61E^ mutation was epistatic to the Δ*luxQ* Δ*cqsS* Δ*vpsS* Δ*cqsR* mutations and restored the colonization defects (>10,000-fold) of the quadruple receptor mutants (Fig 4C). Thus, even though these four receptors contribute to the control of the *V*. *cholerae* QS response to different extents under laboratory conditions, any one of the receptors appears to be sufficient to promote LuxO activation enough to support colonization of mice.

### Multiple sensory inputs maintains the robustness of *V*. *cholerae* QS system

**Fig 5:**
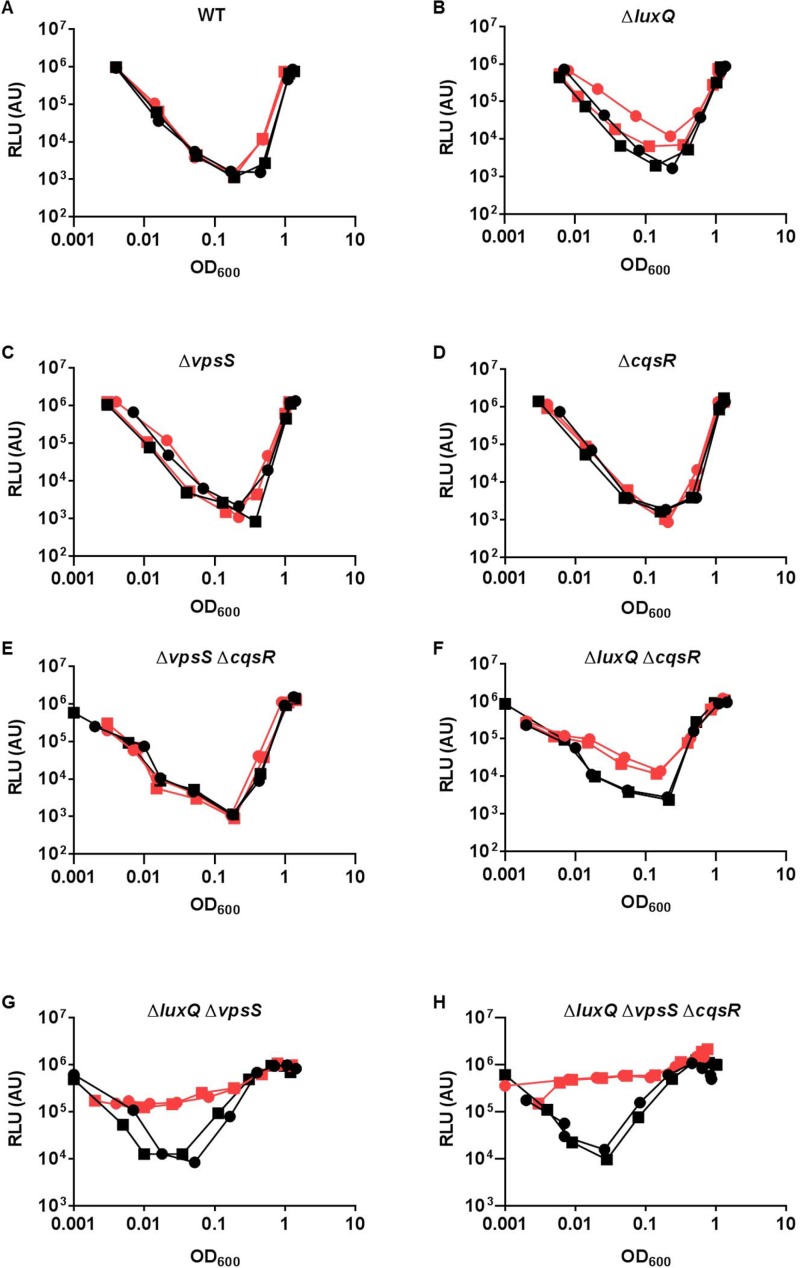
Multiple sensory inputs maintain *V*. *cholerae* QS system robustness. (A-H) The QS response in different *V*. *cholerae* CqsS-expressing strains was measured with a HapR-dependent bioluminescence operon. Normalized light production was measured in different strains in duplicates. RLU denotes relative light units. The genotype of the strain used is listed above each graph. Black lines and symbols represent samples without additional CAI-1. Red lines and symbols represent samples with additional 20 μM CAI-1.

It is curious that *V*. *cholerae* integrates four parallel sensory inputs to activate a common response regulator LuxO, even though a single receptor is sufficient for a QS response (Fig 4B and 4C). We hypothesized that by integrating multiple signals, LuxO activation and the downstream QS response is less sensitive to perturbations from any one of the sensory inputs. To test this idea, we first determined that 2μM of synthetic CAI-1 was sufficient to induce a premature QS response in the triple receptor mutant expressing only CqsS (S5 Fig). Then, we measured HapR-dependent bioluminescence in the presence of surplus CAI-1 (20 μM) in the wild-type and different receptor mutants. Consistent with our prediction, we found that extra CAI-1 did not significantly alter the HapR-dependent bioluminescence profiles of the wild-type or any single receptor mutant missing LuxQ or VpsS or CqsR (Fig 5A–5D). We likewise found that extra CAI-1 did not significantly increase light production in strains expressing CqsS and LuxQ (Δ*vpsS* Δ*cqsR)* (Fig 5E), but addition of CAI-1 slightly, yet reproducibly, increased light production in strains expressing CqsS and VpsS (Δ*luxQ* Δ*cqsR*) (Fig 5F), indicating that inhibition of CqsS kinase activity is compensated for by LuxQ and partially by VpsS (Fig 5E and 5F). In contrast, surplus CAI-1 caused the strains expressing CqsS and CqsR (Δ*luxQ* Δ*vpsS*) to produce light constitutively, indicating that CqsR is not sufficient to compensate for the loss of CqsS kinase activity (Fig 5G). Finally, as expected, strains expressing CqsS alone (Δ*luxQ* Δ*cqsR* Δ*vpsS*) constantly produced light in the presence of surplus CAI-1, as no compensating kinase activity is present (Fig 5H). Thus, functionally redundant receptors, particularly LuxQ and VpsS, render *V*. *cholerae* cells insensitive to surplus CAI-1. These combined results are consistent with the idea that multiple parallel sensory inputs controlling a single LuxO protein are important for resisting perturbations in signal inputs to maintain the robustness of the *V*. *cholerae* QS system.

### VpsS and CqsR activities are regulated by extracellular molecules

**Fig 6:**
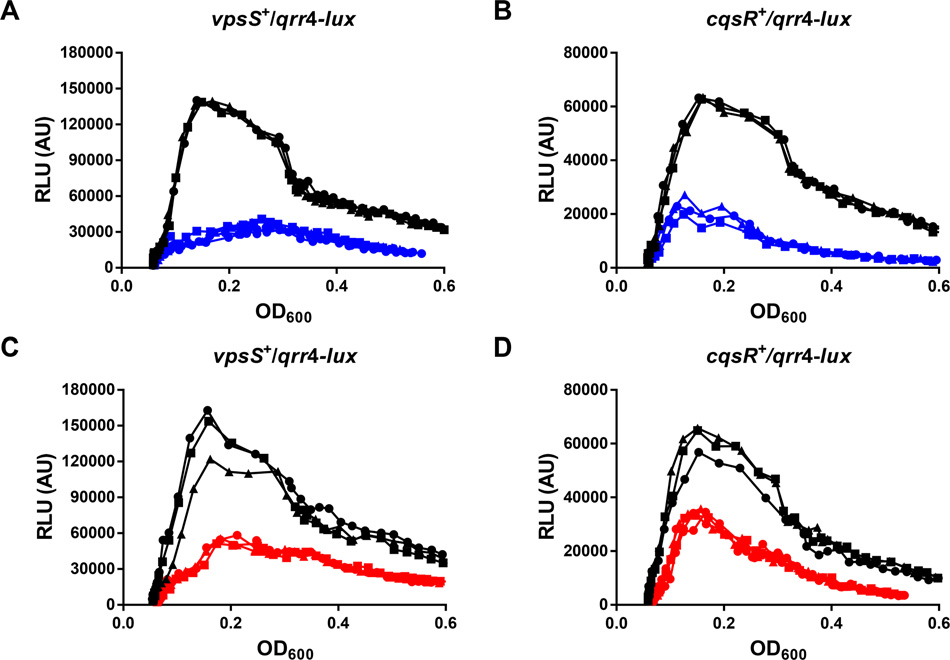
VpsS and CqsR activities are modulated by molecules secreted by *V*. *cholerae*. Qrr4 production in *V*. *cholerae* expressing only VpsS (A and C) or CqsR (B and D) was measured with a *qrr*4-*lux* bioluminescence reporter in the presence and absence of spent culture media harvested from wild-type (A-B) or from the Δ*cqsA* Δ*luxS* mutants (C-D). Normalized light production was measured at least in triplicates. RLU denotes relative light units. Black lines and symbols indicate samples grown in fresh medium. Blue and red lines and symbols indicate samples grown in the presence of 80% (v/v) spent culture medium harvested from the wild-type and the Δ*cqsA* Δ*luxS* mutants, respectively, supplemented with 20% of 5× LB.

To achieve QS regulation, the activities of VpsS and CqsR must be controlled by a cell density dependent mechanism. We reasoned that, similar to CqsS and LuxPQ, the autokinase activities of VpsS and CqsR could both be inhibited by binding to specific molecules that accumulate during cell growth (Fig 1). Therefore, we studied the effects of addition of cell-free spent medium harvested from *V*. *cholerae* HCD cultures on Qrr sRNA expression in the two triple receptor mutants expressing either VpsS or CqsR with a *qrr*4-*lux* reporter. To ensure that any observed regulatory effect from the spent medium was not due to nutrient deprivation after bacterial growth, we replenished any missing ingredients by reconditioning the spent medium (80% v/v) with 20% (v/v) of 5× LB. As expected, when the strains were grown in fresh medium, Qrr4 expression levels were high at LCD and low at HCD (Fig 6A–6D). In contrast, addition of reconditioned spent culture medium decreased LCD Qrr4 production in both strains (Fig 6A and 6B). Qrr4 expression was also repressed by reconditioned spent culture medium harvested from a Δ*cqsA* Δ*luxS* double synthase mutant that cannot make CAI-1 and AI-2 (Fig 6C and 6D), indicating that the signals sensed by VpsS and CqsR are different from the two canonical autoinducers. Addition of reconditioned spent culture medium did not alter the growth rates of these two strains (S6 Fig). Moreover, reconditioned spent medium harvested from LCD (OD_600_ ~ 0.5) *V*. *cholerae* did not alter Qrr4 expression in these two strains (S7 Fig). These combined results suggest that additional molecules other than CAI-1 and AI-2 are made and secreted by *V*. *cholerae* to regulate VpsS and CqsR kinase activities and ultimately control its QS response.

## Discussion

In the current study, we show that the *in vitro* and *in vivo* behaviors of the Δ*luxU* mutant are essentially identical to the Δ*luxO* mutant, therefore suggesting that all the LuxO-activation inputs, including VpsS and CqsR, must shuttle through LuxU to activate LuxO. Indeed, if VpsS and CqsR do not signal through LuxU to activate LuxO, mutants lacking LuxU would behave like mutants lacking CqsS and LuxQ. However, we demonstrate in multiple assays that this was not the case. Therefore, we propose that LuxO, the key QS regulator, is activated by four independent histidine kinase receptors CqsS, LuxQ, VpsS, and CqsR through HPT protein LuxU to control the QS response in *V*. *cholerae* (Fig 1).

Our new model provides additional insights into the *V*. *cholerae* QS signal transduction pathway and explains why *V*. *cholerae* mutants missing the canonical QS receptors CqsS and LuxQ remain proficient in controlling cell density-dependent genes [6]. The influence on LuxO activation of each of the four histidine kinases is not identical; LuxQ is the strongest and CqsR is the weakest activator of LuxO (Fig 4B). Similarly, Yildiz and coworkers previously showed that overexpression of LuxQ and VpsS, but not CqsR and CqsS, increases *vpsL* expression and biofilm formation through a LuxO-dependent mechanism [32]. Surprisingly, our results are in contrast to previous studies of autoinducer synthase mutants in which the QS response is affected more by a Δ*cqsA* mutation than a Δ*luxS* mutation, arguing that CqsS has a stronger impact than LuxQ on *V*. *cholerae* QS [6, 36]. However, it should be noted that our current study was performed under conditions in which autoinducers are produced by *V*. *cholerae* cells at their native levels. Thus, the accumulation rate of each cognate signal in the culture and the signal sensitivity of each receptor could influence the contribution of each receptor to QS control.

Unlike the case in laboratory cultures, CqsS, LuxQ, CqsR, or VpsS alone is sufficient to activate LuxO enough for *V*. *cholerae* to effectively colonize the mouse small intestine (Fig 4C). That is, the loss of three histidine kinase activities has little effect on *V*. *cholerae* colonization of animal hosts, a trait that is strongly dependent on LuxO activation. Perhaps the overall kinase activities of these receptors are substantially stronger in *V*. *cholerae* growing inside an animal than in bacteria growing under laboratory conditions. Additionally, the level of the cognate signals for these receptors could be altered in the host environment such that each receptor maintains a longer period of activation. Alternatively, the level of LuxO activation required to repress HapR-dependent bioluminescence and activate biofilm formation under laboratory conditions is higher than the level required for expression of virulence genes in animal hosts, and that could also explain the difference observed between the *in vitro* and *in vivo* phenotypes. It is interesting that differential contribution from multiple receptor inputs is observed in other microbial signaling pathways. For instance, the sporulation pathway of *Bacillus subtilis* is controlled by five histidine kinases, KinA-E [37–40]. These five receptors participate in the phosphorylation of Spo0F, which in turn activates the key response regulator Spo0A. Although all of these kinases can activate Spo0A, only KinA and KinB can activate Spo0A to a level high enough to trigger sporulation, while KinC and KinD kinases are only capable of initiating entry into stationary phase [40, 41]. Thus, this “many-to-one” configuration has evolved independently in multiple bacterial signal transduction pathways to maintain a specific input-output relationship depending on particular environmental parameters [42].

It is not uncommon for bacterial species to possess multiple QS systems for cell-cell communication. These systems can be wired in different configurations to accomplish specific biological goals [43, 44]. We show here that by using four different receptors in parallel to control the overall QS response, the *V*. *cholerae* QS circuit is built to resist perturbations in external conditions (Fig 5). This circuit architecture could be especially important to maintain synchronous expression of QS genes in the population, and to prevent premature commitment to HCD gene expression. This set up could also be useful for filtering out signal noise caused by analogous molecules present in the environment. It should be noted that a high level of CAI-1 was tested for the sensitivity of the system and *V*. *cholerae* likely will not encounter CAI-1 alone without other autoinducers. However, previous studies showed that molecules with structures drastically different from CAI-1 could inhibit CqsS activity [45], suggesting possibilities for decoy molecules acting alone on a single QS receptor. Such circuitry has been proposed to function as a “coincidence detector” in other QS systems [18, 46]. However, whether the *V*. *cholerae* QS circuit is used for coincidence detection requires further investigation. Indeed, we suspect that not all genes in the *V*. *cholerae* QS regulon display the same regulatory pattern as the HapR-dependent bioluminescence operon, and we predict that a subset of *V*. *cholerae* QS genes could be more sensitive to perturbations. For instance, it has been shown that addition of CAI-1 alone is able to resuscitate viable but non-culturable (VNBC) *V*. *cholerae* [47]. Although these VNBC cells are physiologically distinct from cells cultured under laboratory conditions, these results suggest that a single autoinducer input can trigger differential gene expression in certain *V*. *cholerae* cell types.

The other advantage of using multiple sensory systems is to allow QS bacteria to decipher distinctive information contained within each specific signal. For instance, *V*. *harveyi* detects three autoinducers HAI-1, CAI-1, and AI-2, using LuxN, CqsS, and LuxPQ, respectively, to control its QS response. These circuits are proposed to be used for intra-species, intra-genus, and inter-species communication, respectively [18, 21, 48]. Intriguingly, both VpsS and CqsR are predicted to be capable of detecting small chemical molecules. VpsS is predicted to be cytoplasmic, as it lacks any obvious membrane spanning domain. However, *vpsV*, a gene upstream of *vpsS*, could encode the signal-sensing partner [32]. VpsV carries a FIST domain, which could bind small ligands [49]. CqsR, in contrast, is predicted to be membrane-bound and possess a periplasmic CACHE domain, which is often found in receptors that detect amino acids and other molecules [50–52]. Thus, we speculate that the signals detected by VpsS and CqsR are chemical in nature and contain information that is absent from CAI-1 and AI-2. Although VpsS and CqsR are found predominantly in *Vibrio* species, it remains to be determined if their cognate signals are used for enumeration of species composition or as cell density proxies.

## Materials and Methods

### Strains, media, and culture conditions

All *V*. *cholerae* strains used in this study were derived from C6706str2, a streptomycin-resistant isolate of C6706 (O1 El Tor) [53]. *E*. *coli* S17-1 λ*pir* was used as hosts for plasmids. All strains used in this study are described in S1 Table. *V*. *cholerae* and *E*. *coli* cultures were grown with aeration in Luria-Bertani (LB) broth at 30°C and 37°C, respectively. Unless specified, media was supplemented with streptomycin (Sm, 100 μg/ml), tetracycline (Tet, 5 μg/ml), ampicillin (Amp, 100 μg/ml), kanamycin (Kan, 100 μg/ml), chloramphenicol (Cm, 10 μg/ml) and polymyxin B (Pb, 50 U/ml) when appropriate.

### DNA manipulations and mutant construction

All DNA manipulations were performed using standard procedures. High-fidelity PCR was performed using Phusion DNA polymerase (New England Biolabs). Taq DNA polymerase was used for routine screenings. Oligonucleotide sequences used for PCR, site-directed mutagenesis, and sequencing reactions will be provided upon request. Deletions and point mutations were introduced into the *V*. *cholerae* genome by allelic exchange using the suicide vector pKAS32 [54]. Mutations carried in vector pKAS32 from *E*. *coli* donors were introduced into the *V*. *cholerae* genome by conjugation on LB plates. Transconjugants were selected for by plating on Pb/Amp plates. Subsequent recombinants were selected on LB/Sm (5000 μg/ml) plates, followed by single colony isolation on LB/Sm (5000 μg/ml) plates. Mutant strains carrying the desired mutations were screened and confirmed by PCR. All mutant strains were confirmed by sequencing at the Tufts University Core Facility.

### Infant mouse colonization model

*V*. *cholerae* bacterial cultures were grown aerobically for 16 hr in LB/Sm at 30°C. Mutant strains were then mixed equally with the wild-type Δ*lacZ* strain and approximately 10^6^ colony forming units (CFU) were fed orally to 3- to 5-day-old CD-1 mice (Charles River Laboratories). Prior to infection, infant mice were housed with ample food and water for at least 24 hr and monitored in accordance with the regulations of the Department of Laboratory Animal Medicine at Tufts University School of Medicine. Infected infant mice were sacrificed 24 hr post inoculation and their small intestines were harvested and homogenized. *V*. *cholerae* colonization in the small intestine was measured by plating serial dilutions of intestinal homogenate on LB/Sm/X-Gal plates and enumerating bacterial colonies the next day. Competitive index (CI) was calculated as the ratio of output to input of the mutant strain relative to the wild-type. A minimum of eight infected animals were used to calculate CI. *V*. *cholerae* colonization of the small intestine is presented as a single data point per mouse and data are graphed with the median. If the mutant strains were below the level of detection, it was assumed that there was 1 mutant CFU present at the next lowest dilution of the wild-type sample (indicated by open symbols in the figures).

### HapR-dependent bioluminescence assays

*V*. *cholerae* strains carrying cosmid pBB1 [6], which harbors the heterologous *V*. *harveyi luxCDABE* operon, were first streaked on LB/Tet plates. Individual colonies were then grown aerobically for 16 hr at 30°C in LB/Tet. Cultures were further diluted 1:200 in 20 ml of LB/Tet and grown at 30°C with aeration. OD_600_ (1 ml of culture) and light production (0.1 ml of culture) were measured every 45–60 min for at least 10 hr using a Thermo Scientific Evolution 201 UV-Visible Spectrophotometer and a BioTek Synergy HT Plate Reader, respectively. Light production per cell was calculated from dividing light production by OD_600_. For the assays that determined the effects of surplus CAI-1, a 100 mM CAI-1 stock dissolved in DMSO was diluted to 20 μM in fresh media, DMSO was used as a negative control. For the assays that determined the effects of LuxO overexpression, IPTG was added to the cultures at 100 μM.

### Effect of reconditioned spent culture medium on Qrr4 expression

Spent culture medium was prepared from wild-type *V*. *cholerae* or the Δ*cqsA* Δ*luxS* double synthase mutant. These two strains were grown in LB at 30°C aerobically to HCD (OD_600_ >4). Cells were removed by centrifugation and the supernatants were filtered through a 0.2 μm filter. Filtered cell-free spent culture medium (80%, v/v) was reconditioned by adding back 20% (v/v) 5× LB. As a control, fresh medium was prepared by adding 1× LB (80%, v/v) to 20% (v/v) 5× LB. *V*. *cholerae* mutants expressing only *vpsS* or *cqsR* (*vc1831*) and carrying pBK1003 (P_*qrr*4_-*lux*) [55] were inoculated (1:1000 dilution) into these two media conditions in triplicate and grown in a 96-well microplate at 30°C with aeration. OD_600_ and light production were measured every 30 min for at least 10 hr using a BioTek Synergy HT Plate Reader. Light production per cell was calculated from dividing light production by OD_600_.

### Ethics statement

All animal experiments were done in accordance with NIH guidelines, the Animal Welfare Act, and US federal law. The infant mouse colonization experimental protocol B2013-03 was approved by Tufts University School of Medicine's Institutional Animal Care and Use Committee. The mice were housed in a centralized and AAALAC-accredited research animal facility that is fully staffed with trained husbandry, technical and veterinary personnel.

### Accession numbers (UniProt)

CqsS Q9KM66

LuxP Q9KLK6

LuxQ Q9KLK7

VpsS Q9KS16

CqsR Q9KR16

LuxO Q9KT84

LuxU Q9KT83

AphA H9L4T0

HapR B2CKP3

LuxS Q9KUG4

CqsA Q9KM65

CsrA Q9KUH3

## Supporting Information

S1 Table Bacterial strains used in this study. (PDF)

S1 Fig Effect of *luxO* ^D61E^ mutation on the QS response in a quadruple receptor *Vibrio cholerae* mutant. The QS response in different *V*. *cholerae* mutants was measured with a HapR-dependent bioluminescence operon. Normalized light production was measured in triplicates. RLU denotes relative light units. Blue lines and symbols represent the *luxO* ^D61E^ strain, black lines and symbols represent the Δ*cqsS* Δ*luxQ* Δ*vpsS* Δ*cqsR* strain, and red lines and symbols represent the Δ*cqsS* Δ*luxQ* Δ*vpsS* Δ*cqsR luxO* ^D61E^ strain.
(PDF)

S2 Fig LuxO activation is restored in the quadruple receptor mutant by overexpressing individual QS receptors. The QS response of the quadruple receptor mutants (Δ*cqsS* Δ*luxQ* Δ*vpsS* Δ*cqsR*) expressing CqsS, LuxPQ, VpsS, or CqsR individually was measured with a HapR-dependent bioluminescence operon. Normalized light production was measured in duplicates. RLU denotes relative light units.
(PDF)

S3 Fig Effects of different QS mutations on Qrr sRNA production in *V*. *cholerae*. Qrr4 production was measured with a *qrr*4-*lux* reporter in *V*. *cholerae* mutants as indicated. Normalized light production was measured in triplicates. RLU denotes relative light units.
(PDF)

S4 Fig Effect of *csrA* mutations on Qrr sRNA production in *V*. *cholerae*. Qrr4 expression was measured with a *qrr*4-*lux* reporter in different *V*. *cholerae* mutants as indicated. Normalized light production was measured in triplicates. RLU denotes relative light units.
(PDF)

S5 Fig Induction of premature QS response in the triple receptor mutant expressing only CqsS by additional CAI-1. The QS response of the triple receptor mutant (Δ*luxQ* Δ*vpsS ΔcqsR*) expressing CqsS only was measured with a HapR-dependent bioluminescence operon. Normalized light production was measured in duplicates at OD_600_ ~0.02 with different concentrations of CAI-1 as shown. RLU denotes relative light units.
(PDF)

S6 Fig Growth of *V*. *cholerae* mutants in reconditioned spent culture medium. Bacterial growth was measured by OD_600_ every 30 minutes. Black curves indicate growth in fresh medium. Blue curves indicate growth in the presence of reconditioned spent medium harvested from the wild-type. Red curves indicate growth in the presence of reconditioned spent medium harvested from the Δ*cqsA* Δ*luxS* mutant.
(PDF)

S7 Fig LCD reconditioned spent medium does not affect Qrr4 expression in triple receptor mutants expressing only VpsS or CqsR. Qrr4 expression in triple receptor mutants expressing only VpsS or CqsR was measured with a *qrr*4-*lux* reporter in the presence or absence of 80% (v/v) reconditioned spent medium harvested from wide-type *V*. *cholerae* grown to OD_600_ ~0.5. Normalized light production was measured in triplicates. 20% 5× LB was added to supplement any loss of nutrients. RLU denotes relative light units.
(PDF)
