# Supplementary material for: Quadruple Quorum-Sensing Inputs Control Vibrio cholerae Virulence and Maintain System Robustness
Source: PLoS Pathog. 2015 Apr 15;11(4):e1004837. doi: 10.1371/journal.ppat.1004837 (PMC4398556; doi:10.1371/journal.ppat.1004837)
Supplement: S1 Table — (PDF) [file ppat.1004837.s001.pdf]

SUPPORTING INFORMATION

S1 Table. Bacterial strains used in this study.

**Strain number**	**Relevant genotype**	**Antibiotic resistance(s)**	**Source**
V. cholerae strains			
WN001	WT *Vibrio cholerae* C6706 O1 El Tor	SmR	[1]
WN003	Δ*cqsS* (BH1545)	SmR	[2]
WN004	Δ*cqsS* Δ*luxQ* (BH1569)	SmR	[2]
WN034	Δ*luxQ* pBB1 (BH1589)	TetR	[2]
WN072	Δ*luxO* (SLS349)	SmR	[3]
WN744	Δ*cqsA* Δ*luxS*	SmR	[4]
WN778	*luxO*^{D61E} (SLS340)	SmR	[3]
WN780	Δ*qrr*1-4 (SLS456)	SmR	[5]
WN1171	Δ*luxQ*	SmR	[2]
WN1554	Δ*lacZ*	SmR	[2]
WN3012	*luxO*^{D61A}	SmR	This study
WN3045	Original Δ*luxU* (BH1530)	SmR	[2]
WN3046	Original Δ*luxU* pBB1 (BH1540)	TetR	[2]
WN3053	WT pBK1003	CmR	This study
WN3054	Δ*cqsS* Δ*luxQ* pBK1003	CmR	This study
WN3055	Δ*luxO* pBK1003	CmR	This study
WN3056	*luxO*^{D61E} pBK1003	CmR	This study
WN3059	*luxO*^{D61A} pBK1003	CmR	This study
WN3092	Δ*cqsS* Δ*luxQ* *csrA*::Tn5 pBK1003	CmR	This study
WN3174	Δ*vpsS*	SmR	This study
WN3176	Δ*cqsS* Δ*luxQ* Δ*vpsS*	SmR	This study
WN3189	Δ*cqsS* Δ*luxQ* Δ*vpsS* pBK1003	CmR	This study
WN3201	WT pBB1	TetR	[2]
WN3202	Δ*cqsS* Δ*luxQ* pBB1	TetR	[2]
WN3203	*luxO*^{D61E} pBB1	TetR	This study
WN3204	Δ*vpsS* pBB1	TetR	This study
WN3205	Δ*luxO* pBB1	TetR	[2]
WN3208	Δ*cqsS* Δ*luxQ* Δ*vpsS* pBB1	TetR	This study
WN3350	Δ*cqsS* Δ*luxQ* Δ*cqsR*	SmR	This study
WN3354	Δ*cqsS* Δ*luxQ* Δ*vpsS* Δ*cqsR*	SmR	This study
WN3356	Δ*cqsR*	SmR	This study
WN3357	Δ*cqsS* Δ*luxQ* Δ*cqsR* pBB1	TetR	This study
WN3360	Δ*cqsS* Δ*luxQ* Δ*vpsS* Δ*cqsR* pBB1	TetR	This study
WN3366	Δ*cqsR* pBB1	TetR	This study
WN3369	Δ*vpsS* Δ*cqsR*	SmR	This study
WN3372	Δ*vpsS* Δ*cqsR* pBB1	TetR	This study
WN3383	Δ*cqsS* Δ*luxQ* Δ*cqsR* pBK1003	CmR	This study
WN3385	Δ*cqsS* Δ*luxQ* Δ*vpsS* Δ*cqsR* pBK1003	CmR	This study
WN3391	Δ*vpsS* Δ*cqsR* pBK1003	CmR	This study

WN3557	New Δ*luxU*	SmR	This study
WN3569	New Δ*luxU* pBB1	TetR	This study
WN3627	Δ*cqsS* Δ*vpsS* Δ*cqsR*	SmR	This study
WN3628	Δ*luxQ* Δ*vpsS* Δ*cqsR*	SmR	This study
WN3649	Δ*cqsS* Δ*vpsS* Δ*cqsR* pBB1	TetR	This study
WN3651	Δ*luxQ* Δ*vpsS* Δ*cqsR* pBB1	TetR	This study
WN3671	Δ*cqsS* Δ*luxQ* Δ*vpsS* Δ*cqsR* *luxO*^{D61E}	SmR	This study
WN3724	Δ*cqsS* Δ*luxQ* Δ*vpsS* Δ*cqsR* *luxO*^{D61E} pBB1	TetR	This study
WN3842	Δ*cqsS* Δ*luxQ* Δ*vpsS* Δ*cqsR* pEVS143 pBB1	KanR TetR	This study
WN3843	Δ*cqsS* Δ*luxQ* Δ*vpsS* Δ*cqsR* pEVS143-*cqsS* pBB1	KanR TetR	This study
WN3844	Δ*cqsS* Δ*luxQ* Δ*vpsS* Δ*cqsR* pEVS143-*cqsR* pBB1	KanR TetR	This study
WN3847	Δ*cqsS* Δ*luxQ* Δ*vpsS* Δ*cqsR* pEVS143-*luxQ* pBB1	KanR TetR	This study
WN3848	Δ*cqsS* Δ*luxQ* Δ*vpsS* Δ*cqsR* pEVS143-*vpsS* pBB1	KanR TetR	This study
WN3862	New Δ*luxU* pEVS143-*luxO*^{G333S} pBB1	KanR TetR	This study
WN3872	New Δ*luxU* pEVS143-*luxO* pBB1	KanR TetR	This study
WN3885	New Δ*luxU* pFED342 pBB1	KanR TetR	This study
WN3938	Δ*luxQ* Δ*vpsS*	SmR	This study
WN3940	Δ*luxQ* Δ*cqsR*	SmR	This study
WN3941	Δ*luxQ* Δ*vpsS* pBB1	TetR	This study
WN3945	Δ*luxQ* Δ*cqsR* pBB1	TetR	This study
WN4053	Original Δ*luxU* pBK1003	CmR	This study
WN4056	Δ*cqsS* Δ*vpsS* Δ*cqsR* pBK1003	CmR	This study
WN4060	Δ*luxQ* Δ*vpsS* Δ*cqsR* pBK1003	CmR	This study
WN4061	New Δ*luxU* pBK1003	CmR	This study
WN4074	Δ*cqsS* Δ*luxQ* Δ*vpsS* Δ*cqsR* *luxO*^{D61E} pBK1003	CmR	This study
E. coli strains			
BH032	S17-1λ*pir* pKAS32-*luxO*^{D61E}	AmpR	[6]
WN008	S17-1λ*pir* pKAS32-Δ*cqsS* (MM1428)	AmpR	[2]
WN009	S17-1λ*pir* pKAS32	AmpR	[7]
WN048	S17-1λ*pir* pBB1	TetR	[2]
WN092	S17-1λ*pir* pEVS143	KanR	[8]
WN479	S17-1λ*pir* pFED342	KanR	[9]
WN776	S17-1λ*pir* pKAS32-Δ*luxO* (MM1190)	AmpR	[2]
WN829	S17-1λ*pir* pEVS143-*cqsS*	KanR	This study
WN1438	S17-1λ*pir* pKAS32-Δ*lacZ*	AmpR	[2]

WN2608	S17-1λ*pir* pEVS143-*luxO*	KanR	[10]
WN3013	S17-1λ*pir* pKAS32-*luxO*^{D61A}	AmpR	This study
WN3043	S17-1λ*pir* pBK1003 *(*P$_{qrr4}$*-lux)*	CmR	[9]
WN3167	S17-1λ*pir* pKAS32-Δ*vpsS*	AmpR	This study
WN3342	S17-1λ*pir* pKAS32-Δ*cqsR*	AmpR	This study
WN3426	S17-1λ*pir* pKAS32-Δ*luxQ* (MM1134)	AmpR	[2]
WN3427	S17-1λ*pir* pKAS32-Δ*luxU* (Original construct)	AmpR	[2]
WN3540	S17-1λ*pir* pKAS32-Δ*luxU* (New construct)	AmpR	This study
WN3705	S17-1λ*pir* pEVS143-*cqsR*	KanR	This study
WN3768	S17-1λ*pir* pEVS143-*luxQ*	KanR	This study
WN3820	S17-1λ*pir* pEVS143-*vpsS*	KanR	This study
WN3854	S17-1λ*pir* pEVS143-*luxO*^{G333S}	KanR	This study

References

1. Thelin KH, Taylor RK (1996) Toxin-coregulated pilus, but not mannose-sensitive hemagglutinin, is required for colonization by *Vibrio cholerae* O1 El Tor biotype and O139 strains. Infect Immun 64: 2853-2856.
2. Miller MB, Skorupski K, Lenz DH, Taylor RK, Bassler BL (2002) Parallel quorum sensing systems converge to regulate virulence in *Vibrio cholerae*. Cell 110: 303-314.
3. Hammer BK, Bassler BL (2009) Distinct sensory pathways in *Vibrio cholerae* El Tor and classical biotypes modulate cyclic dimeric GMP levels to control biofilm formation. J Bacteriol 191: 169-177.
4. Hammer BK, Bassler BL (2007) Regulatory small RNAs circumvent the conventional quorum sensing pathway in pandemic *Vibrio cholerae*. Proc Natl Acad Sci U S A 104: 11145-11149.
5. Svenningsen SL, Tu KC, Bassler BL (2009) Gene dosage compensation calibrates four regulatory RNAs to control *Vibrio cholerae* quorum sensing. EMBO J 28: 429-439.
6. Hammer BK, Bassler BL (2003) Quorum sensing controls biofilm formation in *Vibrio cholerae*. Mol Microbiol 50: 101-104.
7. Skorupski K, Taylor RK (1996) Positive selection vectors for allelic exchange. Gene 169: 47-52.
8. Bose JL, Rosenberg CS, Stabb EV (2008) Effects of *luxCDABEG* induction in *Vibrio fischeri*: enhancement of symbiotic colonization and conditional attenuation of growth in culture. Arch Microbiol 190: 169-183.
9. Svenningsen SL, Waters CM, Bassler BL (2008) A negative feedback loop involving small RNAs accelerates *Vibrio cholerae*'s transition out of quorum-sensing mode. Genes Dev 22: 226-238.
10. Ng WL, Perez L, Cong J, Semmelhack MF, Bassler BL (2012) Broad spectrum pro-quorum-sensing molecules as inhibitors of virulence in vibrios. PLoS Pathog 8: e1002767.
