# Supplementary material for: Quadruple Quorum-Sensing Inputs Control Vibrio cholerae Virulence and Maintain System Robustness
Source: PLoS Pathog. 2015 Apr 15;11(4):e1004837. doi: 10.1371/journal.ppat.1004837 (PMC4398556; doi:10.1371/journal.ppat.1004837)

S1 Fig.

Effect of *luxO*^{D61E} mutation on the QS response in a quadruple receptor *Vibrio cholerae* mutant.

The QS response in different *V. cholerae* mutants was measured with a HapR-dependent bioluminescence operon. Normalized light production was measured in triplicates. RLU denotes relative light units. Blue lines and symbols represent the *luxO*^{D61E} strain, black lines and symbols represent the Δ*cqsS* Δ*luxQ* Δ*vpsS* Δ*cqsR* strain, and red lines and symbols represent the Δ*cqsS* Δ*luxQ* Δ*vpsS* Δ*cqsR luxO*^{D61E} strain.

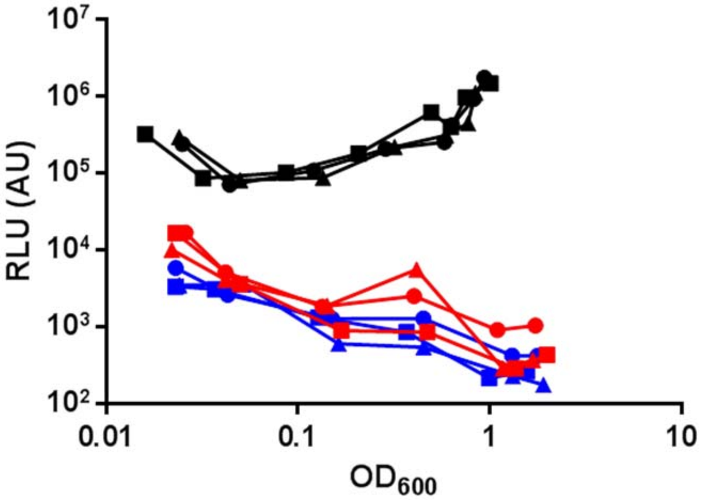

Supplement: S1 Fig — The QS response in different V. cholerae mutants was measured with a HapR-dependent bioluminescence operon. Normalized light production was measured in triplicates. RLU denotes relative light units. Blue lines and symbols represent the luxO D61E strain, black lines and symbols represent the ΔcqsS ΔluxQ ΔvpsS ΔcqsR strain, and red lines and symbols represent the ΔcqsS ΔluxQ ΔvpsS ΔcqsR luxO D61E strain. (PDF) [file ppat.1004837.s002.pdf]
