# Supplementary material for: Quadruple Quorum-Sensing Inputs Control Vibrio cholerae Virulence and Maintain System Robustness
Source: PLoS Pathog. 2015 Apr 15;11(4):e1004837. doi: 10.1371/journal.ppat.1004837 (PMC4398556; doi:10.1371/journal.ppat.1004837)

S2 Fig.

LuxO activation is restored in the quadruple receptor mutant by overexpressing individual QS receptors.

The QS response of the quadruple receptor mutants (Δ*cqsS* Δ*luxQ* Δ*vpsS* Δ*cqsR*) expressing CqsS, LuxPQ, VpsS, or CqsR individually was measured with a HapR-dependent bioluminescence operon. Normalized light production was measured in duplicates. RLU denotes relative light units.

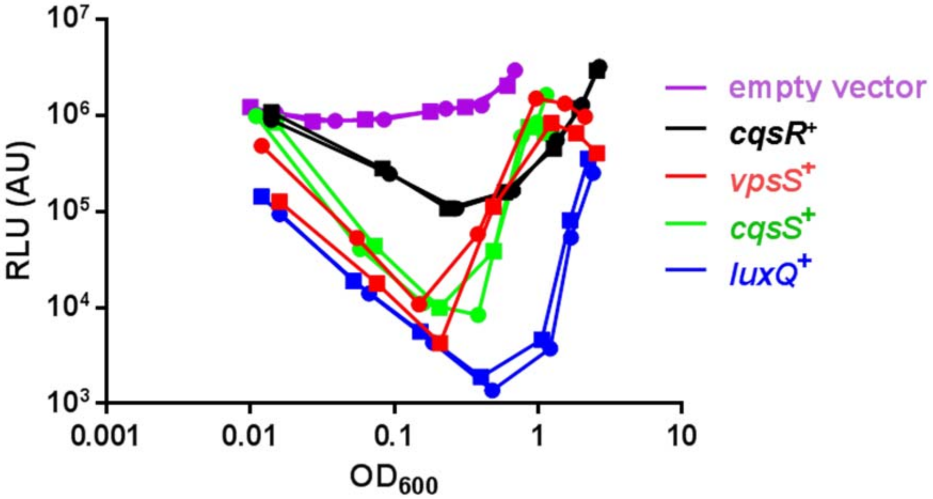

Supplement: S2 Fig — The QS response of the quadruple receptor mutants (ΔcqsS ΔluxQ ΔvpsS ΔcqsR) expressing CqsS, LuxPQ, VpsS, or CqsR individually was measured with a HapR-dependent bioluminescence operon. Normalized light production was measured in duplicates. RLU denotes relative light units. (PDF) [file ppat.1004837.s003.pdf]
