# Supplementary material for: Quadruple Quorum-Sensing Inputs Control Vibrio cholerae Virulence and Maintain System Robustness
Source: PLoS Pathog. 2015 Apr 15;11(4):e1004837. doi: 10.1371/journal.ppat.1004837 (PMC4398556; doi:10.1371/journal.ppat.1004837)

**S3 Fig.**

**Effects of different QS mutations on Qrr sRNA production in *V. cholerae*.**

Qrr4 production was measured with a *qrr*4-*lux* reporter in *V. cholerae* mutants as indicated. Normalized light production was measured in triplicates. RLU denotes relative light units.

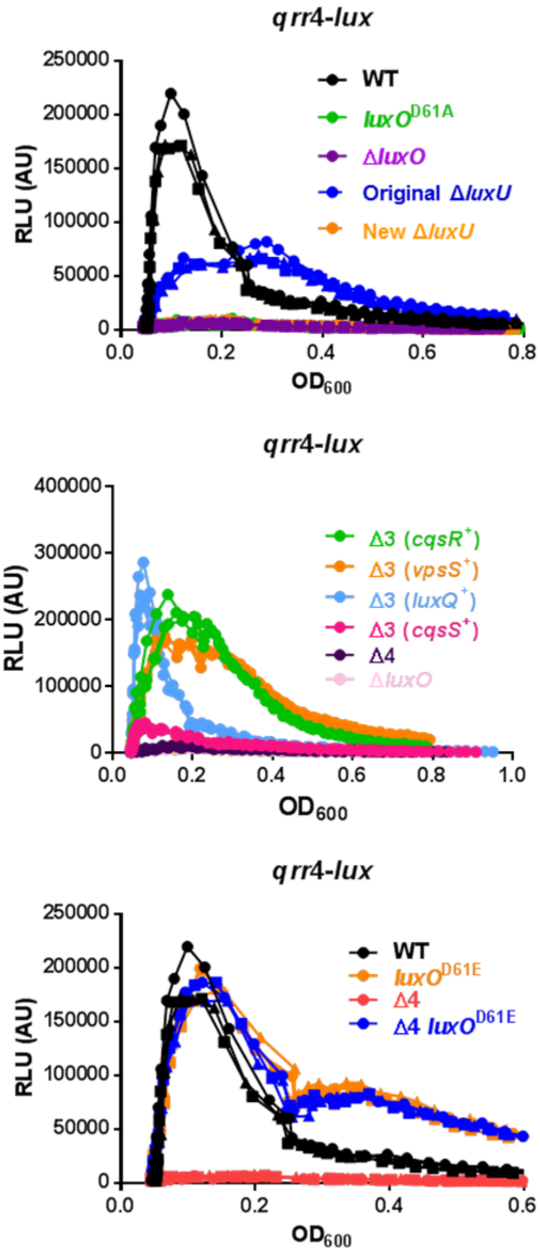

Supplement: S3 Fig — Qrr4 production was measured with a qrr4-lux reporter in V. cholerae mutants as indicated. Normalized light production was measured in triplicates. RLU denotes relative light units. (PDF) [file ppat.1004837.s004.pdf]
