# Supplementary material for: Quadruple Quorum-Sensing Inputs Control Vibrio cholerae Virulence and Maintain System Robustness
Source: PLoS Pathog. 2015 Apr 15;11(4):e1004837. doi: 10.1371/journal.ppat.1004837 (PMC4398556; doi:10.1371/journal.ppat.1004837)

S5 Fig.

Induction of premature QS response in the triple receptor mutant expressing only CqsS by additional CAI-1.

The QS response of the triple receptor mutant (Δ*luxQ* Δ*vpsS* Δ*cqsR*) expressing CqsS only was measured with a HapR-dependent bioluminescence operon. Normalized light production was measured in duplicates at OD_{600} ~0.02 with different concentrations of CAI-1 as shown. RLU denotes relative light units.

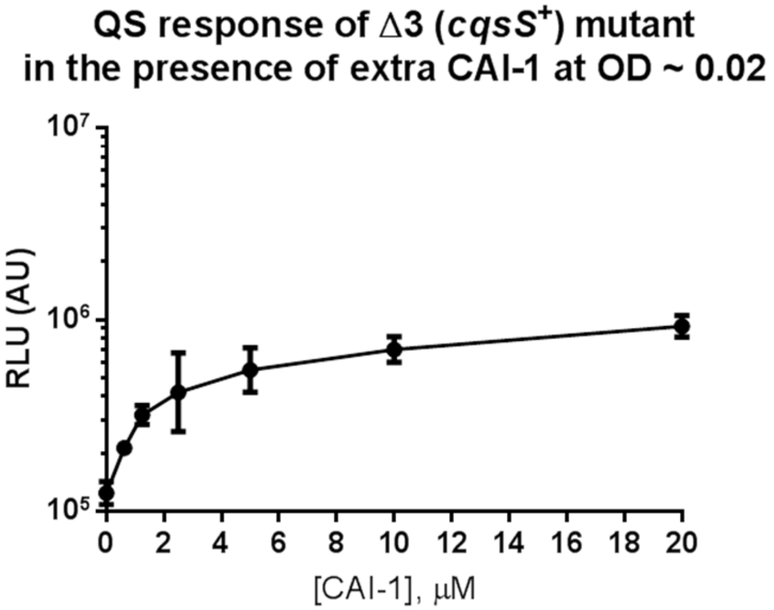

Supplement: S5 Fig — The QS response of the triple receptor mutant (ΔluxQ ΔvpsS ΔcqsR) expressing CqsS only was measured with a HapR-dependent bioluminescence operon. Normalized light production was measured in duplicates at OD600 ~0.02 with different concentrations of CAI-1 as shown. RLU denotes relative light units. (PDF) [file ppat.1004837.s006.pdf]
