# Supplementary material for: Quadruple Quorum-Sensing Inputs Control Vibrio cholerae Virulence and Maintain System Robustness
Source: PLoS Pathog. 2015 Apr 15;11(4):e1004837. doi: 10.1371/journal.ppat.1004837 (PMC4398556; doi:10.1371/journal.ppat.1004837)

**S6 Fig.**

**Growth of *V. cholerae* mutants in reconditioned spent culture medium.**

Bacterial growth was measured by $OD_{600}$ every 30 minutes. Black curves indicate growth in fresh medium. Blue curves indicate growth in the presence of reconditioned spent medium harvested from the wild-type. Red curves indicate growth in the presence of reconditioned spent medium harvested from the Δ*cqsA* Δ*luxS* double synthase mutant.

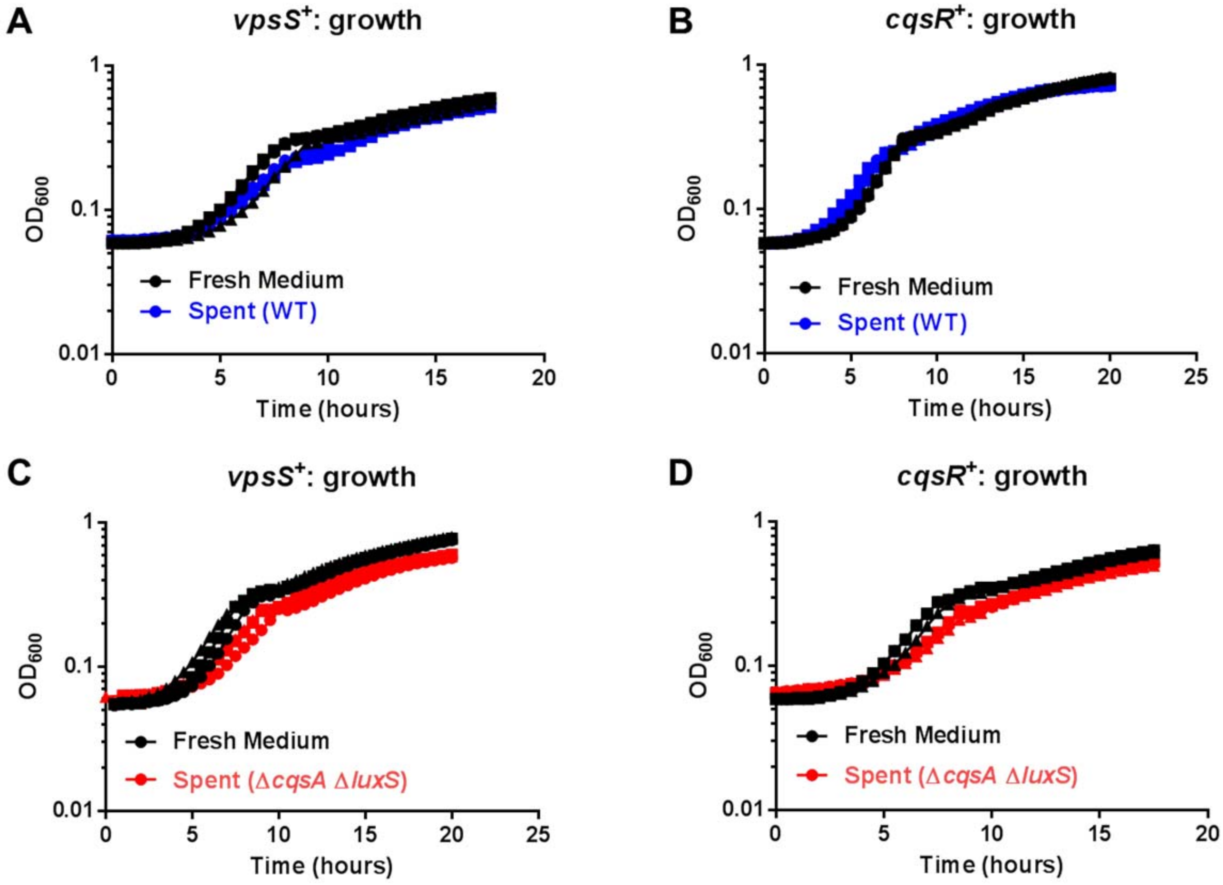

Supplement: S6 Fig — Bacterial growth was measured by OD600 every 30 minutes. Black curves indicate growth in fresh medium. Blue curves indicate growth in the presence of reconditioned spent medium harvested from the wild-type. Red curves indicate growth in the presence of reconditioned spent medium harvested from the ΔcqsA ΔluxS mutant. (PDF) [file ppat.1004837.s007.pdf]
