# Supplementary material for: Quadruple Quorum-Sensing Inputs Control Vibrio cholerae Virulence and Maintain System Robustness
Source: PLoS Pathog. 2015 Apr 15;11(4):e1004837. doi: 10.1371/journal.ppat.1004837 (PMC4398556; doi:10.1371/journal.ppat.1004837)

S7 Fig.

LCD reconditioned spent medium does not affect Qrr4 expression in triple receptor mutants expressing only VpsS or CqsR.

Qrr4 expression in triple receptor mutants expressing only VpsS or CqsR was measured with a *qrr*4-*lux* reporter in the presence or absence of 80% (v/v) reconditioned spent medium harvested from wide-type *V. cholerae* grown to OD_{600} ~0.5. Normalized light production was measured in triplicates. 20% 5× LB was added to supplement any loss of nutrients. RLU denotes relative light units.

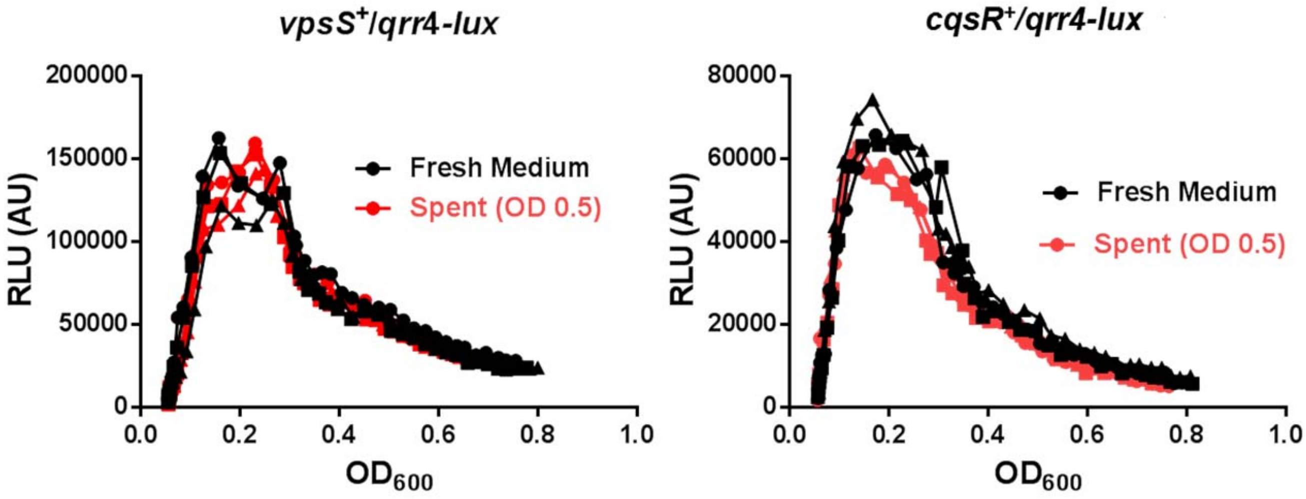

Supplement: S7 Fig — Qrr4 expression in triple receptor mutants expressing only VpsS or CqsR was measured with a qrr4-lux reporter in the presence or absence of 80% (v/v) reconditioned spent medium harvested from wide-type V. cholerae grown to OD600 ~0.5. Normalized light production was measured in triplicates. 20% 5× LB was added to supplement any loss of nutrients. RLU denotes relative light units. (PDF) [file ppat.1004837.s008.pdf]
